# Utility of genetic testing in athletes

**DOI:** 10.1002/clc.23289

**Published:** 2020-01-11

**Authors:** Belinda Gray, Christopher Semsarian

**Affiliations:** ^1^ Agnes Ginges Centre for Molecular Cardiology Centenary Institute New South Wales Australia; ^2^ Faculty of Health and Medical Sciences University of Sydney New South Wales Australia; ^3^ Department of Cardiology Royal Prince Alfred Hospital New South Wales Australia

**Keywords:** athlete, athlete's heart, cardiomyopathy, channelopathy, genetics, sudden cardiac death

## Abstract

Athletes are some of the fittest members of our society, yet paradoxically carry an increased risk of sudden cardiac death (SCD). The athlete's underlying risk of SCD in sports may be increased due to a number of underlying structural, arrhythmic and inherited cardiac conditions (ICCs). There are also physiological adaptations, which occur in the cardiovascular system in athletes as a result of high‐level athletic activity and may be misinterpreted as pathology. Differentiation of “athlete's heart” from heart disease may be challenging due to the effects of exercise on the electrical and structural cardiac remodeling. Features such as prolongation of the QT interval, left ventricular hypertrophy and cavity dilatation, create significant overlap between physiology and inherited channelopathies and cardiomyopathies. Most inherited cardiac conditions have an underlying genetic basis to disease and genetic testing in an athlete can have diagnostic, prognostic and therapeutic implications, including guiding exercise recommendations. Therefore, genetic testing can be a useful diagnostic tool when used carefully and appropriately by a trained cardio‐genetics expert.

## 1 INTRODUCTION

Despite being some of the fittest individuals in society, athletes paradoxically carry an increased risk of sudden cardiac death (SCD) when compared to sedentary individuals with the same cardiac disease.^1^ The prevalence of young sudden cardiac death in the general population is 1.3/100000 individuals aged 1‐35 years, while a recent study of screening in young adolescent football players in the UK showed a much higher incidence of SCD of 6.8/100000 in a young athletic population.^2^, ^3^ SCD is the most common cause of mortality in an athlete during sports.^4^ A number of underlying structural, arrhythmic and inherited cardiac conditions (ICCs) may increase the athlete's risk of SCD in sports. However, there are also physiological adaptations in the athlete, such as prolongation of the QT interval, left ventricular hypertrophy and cavity dilatation that occur as a result of high‐level athletic activity and may be misinterpreted as pathology due to the overlap of these changes with inherited cardiomyopathies and channelopathies.^4^

When evaluating athletes for potential underlying ICCs, including cardiomyopathies and channelopathies, consideration must be given to the genetic basis of a number of these diseases.^5^, ^6^ Genetic studies over the last 30 years have been integral in identifying the gene abnormalities that underpin these diseases. Genetic testing in an athlete can have diagnostic, therapeutic and prognostic value provided that the information is correctly assessed by a cardiogenetics expert in the setting of a specialized multidisciplinary clinic. The yield of genetic testing varies based on the condition for which the athlete is being assessed (from 20%‐30% in dilated cardiomyopathy to 70% in long QT syndrome).^5^ In addition, most ICCs are inherited in an autosomal dominant manner, meaning that first‐degree family members have a 50% chance of also being affected.^7^ It is therefore important that before considering genetic testing in any athlete, that the implications, potential challenges and limitations of the testing are understood by the athlete and their club and that they have undergone comprehensive pre‐ and post‐test genetic counseling.^8^

## 2 GENETIC BASIS TO CARDIAC DISEASE IN ATHLETES

There are a number of physiological adaptations that occur in the heart as a consequence of regular exercise (>4 hours/week). These adaptations are collectively known as “athlete's heart” and can be considered normal in an athlete, therefore not warranting further investigation. However, differentiation of “athlete's heart” from true underlying heart disease can be challenging for the clinician, as a number of these physiological adaptations such as prolongation of the QT interval, left ventricular hypertrophy (LVH) and left and right ventricular dilatation, are also features of inherited channelopathies and cardiomyopathies which may predispose the athlete to dangerous arrhythmias or SCD. Registries have shown that the majority of SCDs in young (<35 years) athletes are due to underlying ICCs including the cardiomyopathies (particularly arrhythmogenic cardiomyopathy [ACM/ARVC], hypertrophic cardiomyopathy and dilated cardiomyopathy [DCM]), and the channelopathies (congenital long QT syndrome [LQTS], catecholaminergic polymorphic ventricular tachycardia [CPVT] and Brugada syndrome [BrS]).^5^, ^9^, ^10^, ^11^, ^12^

When evaluating an athlete for potential underlying ICC, the genetic basis to these conditions must be carefully considered.^5^, ^6^ Molecular studies over the last 30 years have been integral in identifying the particular genetic abnormalities that are the underlying cause of these conditions while rapid technological advances, particularly the advent of next generation sequencing (NGS) technologies, have made genetic testing a readily available tool in the cardiogenetics clinic. Identification of the causative variant allows for predictive (cascade) testing in first‐degree family members due to the autosomal dominant inheritance pattern in most ICCs. Family members who are genotype‐positive require ongoing clinical surveillance and management, while those who are genotype‐negative can be reassured and released from further clinical surveillance.^7^, ^13^

Identification of the causative variant provides benefit to both the athlete under assessment, as well as their family members. Genetic testing has potential utility to assist with diagnosis, prognosis and therapeutics for the athlete, depending on the particular disease in question (Table 1). For some conditions, such as Brugada syndrome, the utility is fairly limited due to the relatively low yield of approximately 20%, whereas in other conditions such as LQTS, the yield can be as high as 70%. In certain situations genetic testing can be used diagnostically to help differentiate between physiology and pathology such as in assessment of the athlete with a prolonged QT interval > 480 ms.^5^, ^14^ In addition to defining the underlying disease, offering genetic testing to athletes (when appropriate) may help to inform potential management outcomes for the athlete including exercise recommendations and in guiding prognosis.^5^, ^15^ For example in LQTS, different genetic subtypes are often offered differing management strategies, while the overall prognosis and risk of arrhythmic events is known to vary between different LQTS subtypes.^16^

**Table 1:** Current utility of genetic testing in athletic individuals for the most commonly encountered cardiomyopathies and channelopathies (adapted from Ackerman et al and Gray, Papadakis)^5^, ^18^

Disease under investigation in the athlete	Diagnostic Utility	Prognostic Utility	Therapeutic Utility	Impact on exercise recommendations
LQTS	+++	+++	+++	+++
CPVT	+++	+	+	+
HCM	++	+	−	−
ACM	++	++	+	+++

*Note*: – no utility, + limited utility, ++ utility in some athletes, +++ clear utility in most athletes. Impact on exercise recommendations derived from Pelliccia et al 2019 EAPC Recommendations^36^ and Ackerman et al 2015 AHA/ACC Recommendations.^17^

In order to improve the yield of genetic testing in an athlete, it is integral to ensure the athlete has undergone a comprehensive clinical evaluation first and that the athlete has a clearly defined phenotype. It is also relevant whether the athlete has a known family history of disease, as the yield is much higher in those with a family history, that is, a higher pre‐test probability. Finally, we would recommend only genes, which have strong supporting evidence to be a cause of the phenotype in question be tested, in order to maximize clinically actionable yield and minimize background noise and variants of uncertain significance (VUS), with pre‐ and post‐test genetic counseling.^5^, ^7^ In an athlete, especially a professional athlete, the diagnosis of an underlying ICC has implications for their future sporting career and therefore it is very important the athlete, their family and any other relevant bodies are included in a shared‐decision model.^17^

## 3 INHERITED CARDIAC CONDITIONS AND INDICATIONS FOR GENETIC TESTING IN ATHLETES

**Figure 1:**
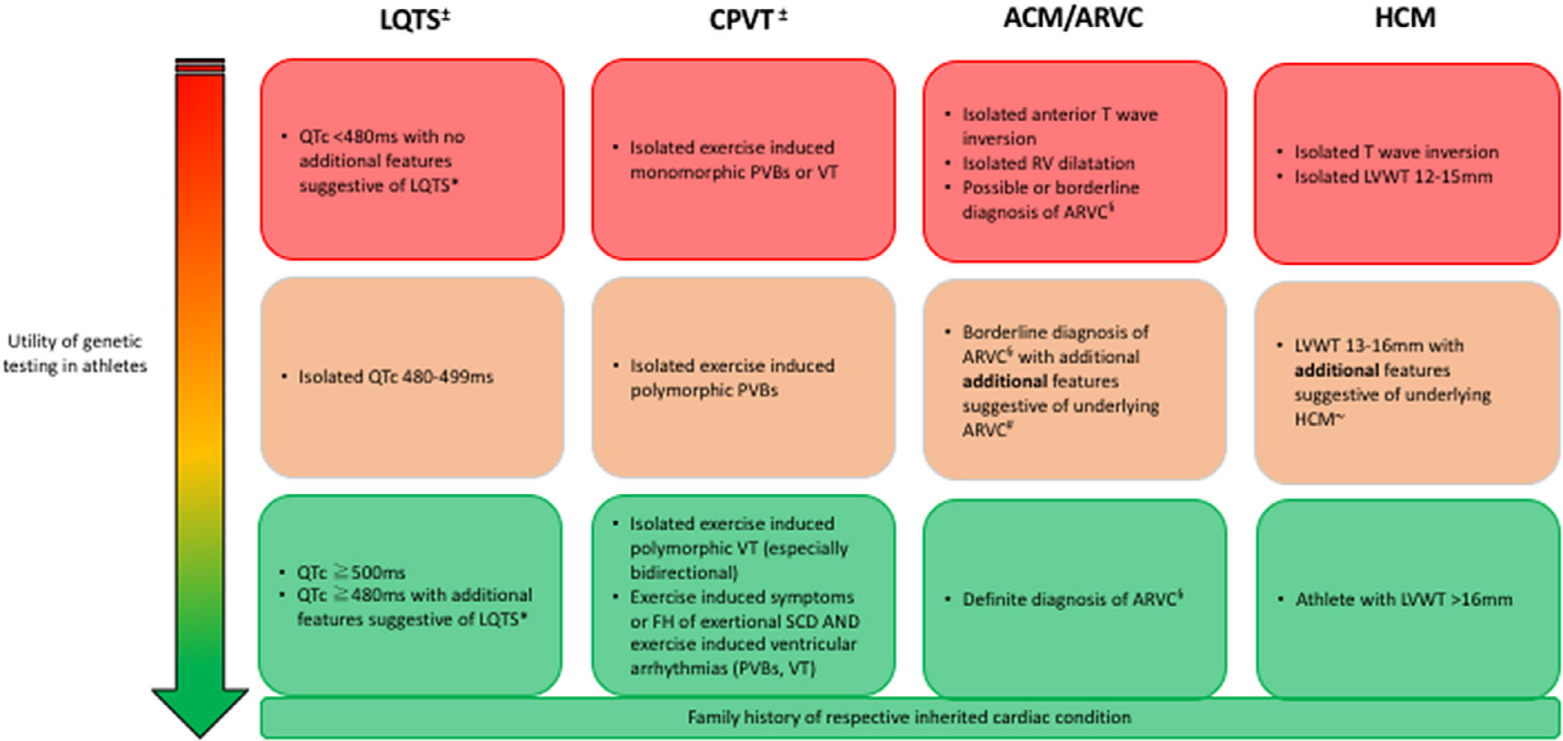
Utility of genetic testing in athletes suspected of an underlying inherited cardiac condition (Modified from Gray, Papadakis 2019).^18^ Red: Not recommended, Orange: May be considered, Green: Should be considered. Key: LQTS‐ Long QT Syndrome, CPVT‐ Catecholaminergic Polymorphic Ventricular Tachycardia, ACM‐ Arrhythmogenic Cardiomyopathy, HCM‐ Hypertrophic Cardiomyopathy, QTc‐ corrected QT interval, PVB‐ premature ventricular beats, VT‐ ventricular tachycardia, LVWT‐ left ventricular wall thickness, SCD‐ sudden cardiac death. ^**±**^ Structural heart disease, coronary artery disease and electrolyte abnormalities should be excluded. * Symptoms, documented polymorphic arrhythmias, paradoxical prolongation of the QT interval during exercise, T‐wave alternans, T wave notching, congenital deafness, family history of unexplained SCD.^19^^§^ According to the 2010 Task Force criteria, excluding genetic testing result.^20^^#^ High propensity to ventricular arrhythmias (eg, high burden of PVCs from RVOT or multifocal PVCs on holter), symptoms (eg, syncope), family history of SCD in a first‐degree relative under the age of 40 years, presence of scar on MRI, RV structural changes with borderline ECG abnormalities (eg, T‐wave inversion in anterior chest leads). ~ Symptoms (eg, syncope), family history of SCD in a first‐degree relative under the age of 40 years, ECG abnormalities (particularly inferolateral T‐wave inversion), asymmetric hypertrophy, myocardial scar on cardiac MRI, blunted blood pressure response to exercise

While genetic testing can be useful when assessing athletes in selected cases, to differentiate physiological adaptation from underling pathology (or ICC), there are some particular conditions where‐by genetic testing has the highest yield and carries the greatest utility. These are highlighted below, and summarized in Figure 1:

### 3.1 LONG QT SYNDROME

Long QT syndrome (LQTS) is an inherited channelopathy with an estimated prevalence of 1:2000.^21^ LQTS is characterized by the presence of a prolonged QTc after secondary causes for a prolonged QTc such as drugs or electrolyte disturbances have been excluded.^6^ LQTS can also be diagnosed in an individual with an LQTS risk score (modified Schwartz score) >3.5 or when an unequivocally pathogenic variant in a LQTS gene is identified on genetic testing.^6^, ^19^

In LQTS, proceeding to genetic testing is an integral part of the diagnostic pathway. In an athlete with a repeatedly prolonged QTc > 480 ms, genetic testing is indicated in most situations, independent of whether the athlete is symptomatic. Athletes tend to exhibit longer QT intervals compared to the general population and an isolated long QT interval in an athlete may represent the effect of increased vagal tone or delayed repolarization as a result of increased left ventricular mass.^22^ A study of 2000 elite athletes demonstrated that identification of a QTc > 500 ms in an athlete was rare, and these athletes were more likely to have an underlying diagnosis of LQTS.^14^

There are currently 17 genes known to cause congenital LQTS, however more than 90% of cases are due to the main 3 genotypes.^6^, ^23^ LQT1 is caused by loss of function mutations affecting *KCNQ1* the gene encoding for I_Ks_ (slow) channel.^23^ LQT2 is caused by loss of function mutations in *KCNH2* the gene encoding for the I_Kr_ (rapid) channel,^24^, ^25^ While LQT3 is caused by gain of function mutations in *SCN5A*, the gene encoding for I_Na_.^26^

In addition to diagnostic utility in LQTS, genetic testing assists with prognostic and therapeutic decision‐making. There are specific genotype associated triggers for ventricular arrhythmias including strenuous exercise with particular risk identified with swimming (LQT1), loud noise/alarm clocks (LQT2) and sleep (LQT3).^27^ All athletes with a prolonged QTc are offered lifestyle advice such as avoidance of QT prolonging medication and correction of electrolyte disturbances while exercising. Identification of a specific LQTS genotype may also help guide the clinician on particular lifestyle recommendations for affected individuals. Additionally, genotype directed therapies such as betablockers, (particularly nadolol) in LQT1 or mexilitene in LQT3 can significantly reduce the life‐threatening arrhythmic risk to the individual athlete.^16^, ^28^

Genetic testing also assists in giving an athlete and their family a personalized prognostic risk. A recent study highlighted that an individual with LQTS can have their arrhythmic risk determined through a composite of the genotype and the QTc of the individual under assessment. This may vary from a low risk individual (eg, QTc < 460 ms especially LQT1 with an estimated 5‐year risk of <1% even without betablocker therapy) to highest risk individuals (eg, LQT2 and LQT3 with QTc > 550, with an estimated 5‐year risk of life‐threatening arrhythmias of >9% off therapy).^16^

### 3.2 CATECHOLAMINERGIC POLYMORPHIC VENTRICULAR TACHYCARDIA

Catecholaminergic polymorphic ventricular tachycardia (CPVT) is a rare, but highly lethal, inherited channelopathy with an estimated prevalence of 1:10000. CPVT is characterized by adrenergically‐stimulated ventricular tachycardia (typically through physical exertion), in the presence of a structurally normal heart.^6^ A key feature of CPVT is that the individuals have a normal baseline ECG and echocardiogram, and therefore without exercise stress testing the diagnosis can be missed. The diagnostic hallmark is of exercise induced ventricular arrhythmias, particularly bidirectional VT. This condition should always be considered in athletes with exercise‐induced syncope.

As in LQTS, genetic testing can assist in CPVT diagnosis with the most common form due to autosomal dominant mutations in the cardiac ryanodine receptor (*RYR2*) which account for up to 60% of cases.^5^ There are also rarer recessive forms of CPVT due to mutations in other calcium handling genes including cardiac calsequestrin (*CASQ2*).^5^, ^29^ CPVT can be diagnosed in athletes who are found to be carriers of a known clearly pathogenic genetic variant, as well as in family members of an index case who develop premature ventricular contractions (PVCs) during exercise.^6^ Any athlete with a structurally normal heart and exercise‐induced syncope, should be assessed for CPVT and genetic testing considered if there is evidence of exercise induced ventricular tachycardia, especially in the presence of bi‐directional ventricular tachycardia.

### 3.3 ARRHYTHMOGENIC CARDIOMYOPATHY

Arrhythmogenic cardiomyopathy (ACM) is an umbrella term, which encompasses an arrhythmogenic heart muscle disease not due to valvular, ischaemic or hypertensive heart disease. The underlying causes for ACM include genetic, systemic and inflammatory disorders, however the key feature is the predominance of arrhythmia in the presentation.^30^ The predominant form of genetic ACM is arrhythmogenic right ventricular cardiomyopathy (ARVC), a disease characterized by fibro‐fatty infiltration of the ventricular myocardium and with a prevalence of approximately 1:2000. Recent post‐mortem data has shown left ventricular histopathological involvement in 87% of cases.^9^ ARVC is diagnosed through a composite scoring system, which constitute the ARVC Task Force Criteria, with a recent HRS consensus statement highlighting the increased challenges in diagnosing left ventricular dominant disease due to the overlap with dilated cardiomyopathy.^20^, ^30^

ARVC is typically due to mutations in desmosomal protein genes including *PKP2*, *DSC2*, *DSG*, *DSP*, and *JUP*.^31^ Left‐sided and biventricular disease often overlaps with dilated cardiomyopathy (DCM) and genetic causes include *LMNA*, *PLN*, *DSP* and *FLNC*.^30^ Identification of a pathogenic variant constitutes a major task force criterion, which may upgrade a suspected case from a possible or borderline to a definite diagnosis.^20^ ACM/ARVC has consistently been shown to be an important cause of SCD in athletes due to ventricular arrhythmias provoked by exercise.^32^ Additionally, the arrhythmic phenotype has been shown to predate the cardiomyopathy phenotype, with one study showing SCD due to ACM was 16 times more common in athletic individuals compared with non‐athletes, often with no prior symptoms.^9^

It is important to highlight that many high‐level athletes have right ventricular structural changes due to physiological adaptation to exercise. However, there are some key clinical features, which have been shown to be more commonly associated with underlying ARVC rather than athletic adaptation. These include symptoms (eg, syncope), family history of SCD, high burden of premature ventricular contractions [PVCs (>1000)] or sustained ventricular arrhythmias, abnormal signal‐averaged electrocardiogram (SAECG) and abnormal scar on CMR (cardiac magnetic resonance) imaging.^33^ Therefore, genetic testing is usually indicated in athletic individuals with a high‐index of clinical suspicion for ARVC such as those with right ventricular structural abnormalities and the presence of the other high‐risk features listed in Figure 1. In athletes with right ventricular structural change in isolation genetic testing is usually not indicated.

Evidence suggests that athletic individuals with ARVC develop symptoms earlier and have a higher prevalence of ventricular arrhythmias, compared with non‐athletes.^34^ Even those athletes who are gene carriers with no overt ARVC phenotype (genotype‐positive phenotype‐negative individuals) have higher disease penetrance with earlier presentation of overt phenotype, more ventricular arrhythmias and more rapid progression to heart failure.^35^ Therefore first degree family members of a genotype positive ARVC case should proceed to genetic testing in order to identify those individuals within the family who are genotype‐positive who are now recommended to avoid competitive sports as of the most recent guidelines.^36^

### 3.4 HYPERTROPHIC CARDIOMYOPATHY

Hypertrophic cardiomyopathy (HCM) is characterized by left ventricular hypertrophy (LVH) in the absence of loading conditions such as aortic stenosis or hypertension and has a prevalence of at least 1 in 500 individuals.^37^ HCM is associated with pathogenic variants in at least 11 genes, typically of cardiac sarcomere proteins including *MYBPC3* and *MYH7*. Athletic individuals may develop significant hypertrophy as part of physiological adaptation to exercise, however a left ventricular wall thickness (LVWT) of >16 mm is very rare, especially in white athletes, and more likely to reflect a diagnosis of HCM. In these individuals, genetic testing should be considered.^36^, ^38^ In athletes with moderate LVH (eg, LVWT 13‐16 mm) in association with other features suggestive of an underlying diagnosis of HCM for example, inferolateral t‐wave inversion, family history, asymmetric LVH, presence of myocardial scar (late gadolinium enhancement [LGE]) on CMR imaging, presence of non‐sustained ventricular tachycardia, or a blunted blood pressure response to exercise, proceeding to genetic testing should also be considered (Figure 1). In these cases, the yield of genetic testing is lower, especially in the absence of a family history however, identification of an actionable variant in a sarcomere gene can help to differentiate the diagnosis. Genetic testing for *PRKAG2* should be considered in athletes with LVH and co‐existent pre‐excitation on the ECG.^39^

The presence of T‐wave inversion on the ECG of an athletic individual can indicate underlying cardiomyopathy, particularly in white athletes with inferolateral t‐wave inversion. However Sheikh et al showed comprehensive clinical evaluation provides significantly greater diagnostic utility (20%) than genetic testing (10%) at a third of the cost. Only athletes with lateral T‐wave inversion were found to have actionable genetic variants with the highest yield of 14% in white athletes with lateral T‐wave inversion. Therefore, the use of genetic testing in this group is usually not indicated.^40^

## 4 OTHER CONSIDERATIONS

**Figure 2:**
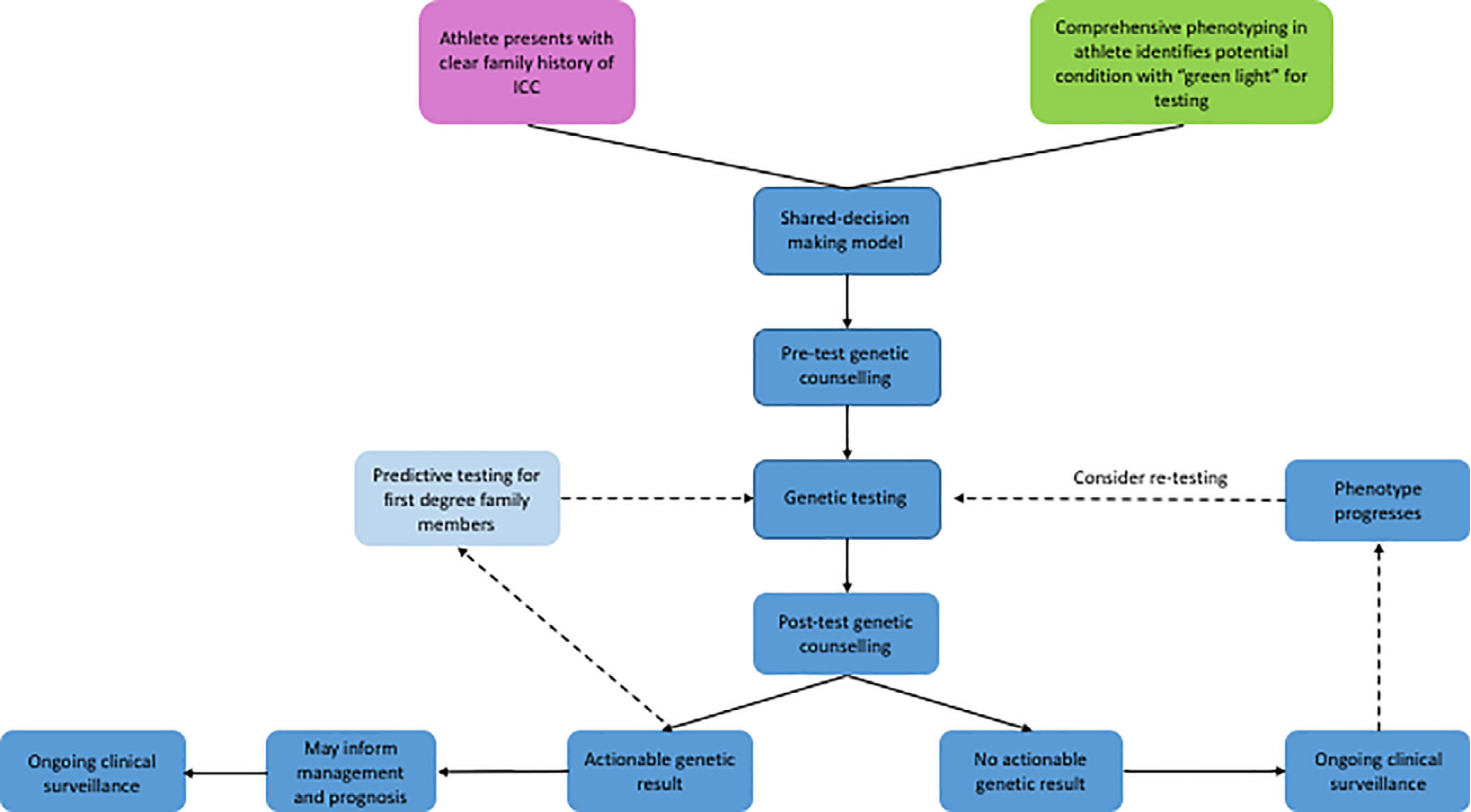
Summary flow diagram outlining the genetic testing process

There are often significant ethical and legal factors for any athlete considering genetic testing that must be carefully considered prior to proceeding. The sporting organization may have specific legal requirements and there may be insurance ramifications for the athlete and their club or organization. However, there are potential benefits in identification of a particular genetic result, including initiating life‐saving medication such as beta‐blockers, which may ultimately facilitate safe “return to play” for the athlete.^17^, ^41^ In addition, given the autosomal dominant genetic basis to most cardiomyopathies and channelopathies, there are important implications for first‐degree family members of the athlete which also require consideration. Therefore, before any athlete proceeds with genetic testing pre‐ and post‐test genetic counseling by an experienced cardiac genetic counselor is essential (Figure 2).^7^, ^8^

## 5 CONCLUSIONS

Genetic testing in an athlete can provide useful diagnostic, therapeutic and prognostic value provided that the testing is performed and the findings interpreted by a cardiogenetics expert in the setting of a specialized multidisciplinary clinic. Identification of actionable variants in LQTS, ARVC and CPVT genes has important implications for management and prognosis for the athlete, as well as helping to inform exercise prescription. However, it is very important that genetic testing should only proceed in athletes following comprehensive clinical phenotyping, when there is a high index of suspicion of an ICC, or in the presence of a family history. Appropriate pre‐ and post‐test genetic counseling is essential. Before proceeding to genetic testing in any athlete, it is critical that the athlete, their family and their club or sporting organization understand the implications, limitations and difficulties of the testing, so that an informed decision is made in a shared decision‐making model of care.
